# Use of *Vitex agnus-castus* in patients with menstrual cycle disorders: a single-center retrospective longitudinal cohort study

**DOI:** 10.1007/s00404-023-07363-4

**Published:** 2024-02-23

**Authors:** Martina Höller, Hubert Steindl, Dimitri Abramov-Sommariva, Julia Kleemann, Alexey Loleit, Christoph Abels, Petra Stute

**Affiliations:** 1 Bionorica SE, Kerschensteinerstr. 11–15, 92318 Neumarkt, Germany; 2 Amstelveenseweg 122A2, 1075 XL Amsterdam, The Netherlands; 3 Department of Obstetrics and Gynecology, Inselspital Bern, Friedbühlstrasse 19, 3010 Bern, Switzerland

**Keywords:** Dysmenorrhea, Mastalgia, Mastodynia, Cyclodynon, Agnucaston, Mastodynon

## Abstract

**Purpose:**

To evaluate clinical characteristics, quality of life (QoL) and effectiveness in patients with menstrual cycle disorders (MCDs) including abnormal uterine bleeding, dysmenorrhea and mastodynia/mastalgia related to premenstrual syndrome taking the *Vitex agnus-castus* (VAC) products Cyclodynon® or Mastodynon® in a real-world setting.

**Methods:**

A single-center retrospective longitudinal cohort study (3 ± 1 months), using data obtained from healthcare data archive and telephone interviews. The main study variables were changes in bleeding, menstrual pain, breast tenderness and patients’ QoL.

**Results:**

Data from 1700 women with a mean age of 30.2 years (± 6.3) were analyzed. The most common MCDs were dysmenorrhea (43.8%) and mastodynia/mastalgia (21.1%). Three-month treatment with VAC extract substantially decreased the percentage of patients with irregular cycle (from 9.1% to 0.1%) and breast tenderness (from 39.9% to 0.8%). Improvement in bleeding intensity, frequency and menstrual pain was experienced by 83.4%, 79.2%, and 85.2% of the patients, respectively. When analyzed by disease category, these parameters improved in almost all dysmenorrhea patients, while they improved to a lesser extent in mastodynia/mastalgia patients. QoL improved in all aspects, but was reported by a higher proportion of dysmenorrhea patients compared to mastodynia/mastalgia patients. Treatment was overall well tolerated with a favorable safety profile.

**Conclusion:**

These real-world data demonstrate the effectiveness of the VAC-containing products Cyclodynon® and Mastodynon® in the three-month treatment of MCDs, with a pronounced improvement in key disease symptoms and QoL. Intriguingly, while QoL was generally greatly improved, the response to VAC therapy varied depending on the type of underlying MCD.

**Supplementary Information:**

The online version contains supplementary material available at 10.1007/s00404-023-07363-4.

## What does this study add to the clinical work

**Table:** 

The findings of this real-world data study confirm the benefit of Cyclodynon® and Mastodynon® in the treatment of MCDs such as AUB, dysmenorrhea or mastodynia/mastalgia related to PMS. Treatment with 4 mg-VAC-containing products was associated with a normalization of the menstrual cycle, reduction of menstrual pain and breast tenderness as well as an improvement in the women’s QoL and daily functioning.	

## Introduction

During the reproductive life span there are several medical conditions associated with the menstrual cycle. Menstrual cycle disorders (MCDs) comprise abnormal uterine bleeding (AUB), pain associated with menstrual cycle (dysmenorrhea) and premenstrual syndrome (PMS) including cyclic breast pain (mastodynia/mastalgia). MCDs affect women of childbearing age worldwide with a prevalence of 30–70% and represent a significant burden by worsening quality of life (QoL) [1].

The term AUB covers a spectrum of irregularities of the menstrual cycle involving abnormal bleeding from the uterine corpus in terms of frequency, regularity, duration and volume [2]. Menstrual cycle regulation is based on a complex interplay of the hormonal axis from the hypothalamus via the pituitary gland to the ovary. From the complex interplay of different hormone concentrations which are controlled by precisely regulated feedback mechanisms, it can be easily concluded that imbalances in the reciprocal interplay of hormones must be reflected in disturbances of the menstrual cycle [3, 4].

Dysmenorrhea is defined as recurrent, crampy lower abdominal pain that occurs during menses due to the shedding of the lining of the uterus, which leads to a recurrent process of inflammation and wound healing [5, 6]. Primary dysmenorrhea usually occurs within 6–24 months after the onset of menarche and the associated pain occurring before or at the onset of menstrual flow typically lasts eight to 72 hours [5]. Other associated symptoms may include nausea, vomiting, diarrhea, fatigue, weakness, fainting, headache [7]. The most widely accepted explanation for the pathogenesis of primary dysmenorrhea is the overproduction of uterine prostaglandins. Specifically, prostaglandins PGF_2_α and PGE_2_ are involved in myometrial contractions, vasoconstriction and hypersensitization of pain fibers, ultimately leading to pain [5]. The production of prostaglandins is regulated by several factors, one of which is progesterone. The drop in progesterone levels prior to menstruation indirectly increases the generation of prostaglandins [5]. Of note, the intensity of the menstrual pain is directly proportional to the amount of prostaglandins released [8].

Another MCD is PMS which includes various physical and psychological symptoms that occur during the luteal phase of the female menstrual cycle and disappear with the onset of menstruation in contrast to dysmenorrhea [9, 10]. Besides common symptoms like mood swings, constipation, sleep problems and headache/migraine, mastalgia/mastodynia (painful/tender breasts) is among the most frequent complaints of women suffering from PMS [10]. The prevalence of this cyclic breast pain has been reported to range from 30 to 69% [11]. There are several hypotheses of PMS etiology one of which is a relative hyperprolactinemia with prolactin serum concentrations remaining within the normal reference range [12, 13].

Many MCDs are already present in adolescence. In 10–15% of patients, MCDs persist from menarche until the age of active reproduction and the prevalence of dysmenorrhea reaches 60–90% in this stage of life [14]. MCDs require long-term treatment, making safety an essential feature of the medication used.

*Vitex agnus-castus* L. (VAC), the fruit of the sacred Vitex, also called chasteberry, chaste berry or monk’s pepper, has a long tradition in the treatment of MCDs [15, 16]. The clinical pharmacological effects of VAC extract are not clear, yet, but supposed to be due to a dopaminergic activity in the hypothalamic-pituitary–gonadal axis, leading to reduced prolactin secretion and potentially alleviating symptoms of PMS and associated mastalgia/mastodynia [12, 13, 17–19].

Cyclodynon® (trade name of Agnucaston® in Russia) and Mastodynon® are commonly used VAC products in gynecological practice. Both products contain the VAC extract BNO 1095 in the same allopathic dosage (4 mg). Mastodynon® in addition contains five herbal substances in a homeopathic dilution (cyclamen, tiger lily, ignatius bean, blue cohosh and blue flag). Both products are approved for the treatment of MCDs, PMS, and also mastodynia and are available as tablets and oral drops.

Several clinical studies of VAC products have demonstrated a high potential to relieve MCDs associated symptoms such as menstrual cycle irregularities, painful menstrual bleeding and menstrual-related migraine [20–24]. Randomized clinical trials (RCTs) investigate the efficacy of a drug under standardized conditions. But even if a drug is highly effective in RCTs, it may be less effective in the real world as effectiveness in routine clinical practice depends on a variety of factors, such as diagnostic accuracy, physician and patient compliance and the country’s healthcare system [25]. Real-world data are therefore not only essential for assessing effectiveness of a medicine following market authorization but also for understanding of patient health and the impact of medicines on patient and system outcomes in routine settings. This observational retrospective longitudinal study over 3 months aimed to evaluate the demographics, gynecological status, clinical characteristics, QoL and treatment effectiveness of patients with MCDs who used Cyclodynon® and Mastodynon® in routine clinical practice.

## Methods

### Study design

This was a single-center, retrospective, longitudinal, observational cohort study of women aged 12–50 years who were prescribed Cyclodynon® or Mastodynon® for the treatment of MCDs in routine clinical practice. The analysis period was 3 (± 1) months after first prescription within the pre-established index period from 1 January 2018 to 30 July 2021; therapy may have been continued afterwards. Both Cyclodynon® and Mastodynon® contain 4 mg of the BNO extract 1095. The study was conducted at the State Budgetary Institution of Health of The Novosibirsk Region “City Clinical Polyclinic No. 14” (Novosibirsk, Russia).

Data were collected retrospectively by evaluation of medical records (main data source) of the eligible patients in the healthcare data archive. The respective anonymized (de-identified) data on medical history and the results of clinical examinations were entered by the investigators into the electronic Case Report Form using a validated electronic data capture system (Data MATRIX GmbH, Germany). If the data in the medical records were incomplete and less than 6 months had passed since the last day of treatment, the investigator conducted a telephone interview with the patient (additional data source).

Extracted data for baseline characteristics included demography, anthropometry, medical history and concomitant diseases, gynecological and obstetric history, duration and type of MCD (AUB, dysmenorrhea or mastodynia/mastalgia related to PMS), prescription of Cyclodynon® and Mastodynon® and concomitant medication. Social and financial status was assessed by the physician on a three-item scale (low, medium, high) for each patient. Gynecological status including the presence of breast tenderness (yes, no, unknown) were documented at baseline and after treatment. Menstrual changes during the observation period were assessed after treatment including bleeding length, bleeding intensity, bleeding frequency, and pain during menstruation (improvement, negative, without change) as well as the incidence of additional bleeding episodes or amenorrhea. In addition, QoL parameters were collected, including sleep quality, stress symptoms, migraine symptoms, headache, acne, constipation, stomach complaints, changes in concentration, mood swings, depression symptoms and libido. These parameters were examined for their presence (yes, no, unknown) or status (satisfactory, unsatisfactory for sleep; decreased, increased, not changed for libido) at baseline as well as for changes (improvement, negative, without changes, unknown) after treatment. Periods of sick leave or reduced daily functioning due to the MCD were collected at baseline and after treatment.

### Study population

Women aged 12–50 years who were prescribed Cyclodynon® or Mastodynon® for an index event registered in the analyzed period for one of the following indications were included into the study: amenorrhea, oligomenorrhea, polymenorrhea, hypomenorrhagia, menorrhagia, hypermenorrhagia, menometrorrhagia, metrorrhagia, intermenstrual bleeding, dysmenorrhea and mastodynia/ mastalgia related to PMS (cyclic breast pain).

### Statistical analysis

The SPSS 26.0 (SPSS Inc., USA) statistical software was used for data analysis. Descriptive statistics for quantitative variables include the mean, standard deviation (SD), median, first and third quartiles, minimum and maximum values, and number of valid observations (*n*). Qualitative parameters are presented as frequencies and proportions. 95% CIs around the point estimate are also presented, if applicable. All endpoints and all statistical analyses were defined in a project plan prospectively. Data management was carried out following an a priori defined data management plan and an a priori defined data validation plan. A local ethics committee (St. Petersburg) approved this prospectively planned data collection.

## Results

### Baseline characteristics of study population

In this study, data from the medical records of 1700 eligible women were analyzed. Demographic, anthropometric, and social characteristics of the study population are shown in Table 1. The mean age of women was 30.2 ± 6.3 years. The age group of 12–17 years was represented by only four patients. Most women were married (85.5%), working (69.1%) and had a medium social/financial status (88.4%), 40.1% were overweight (BMI 25–29.9 kg/m^2^) and 1.8% were obese (BMI ≥ 30 kg/m^2^).

**Table 1:** Socio-demographic and anthropometric characteristics of the study population

Parameter	Study population (*N* = 1700)
Age, years, mean ± SD	30.2 ± 6.3
Age subgroups, years	
12–17, *n (%)*	4 (0.2)
18–29, *n (%)*	821 (48.3)
30–39, *n (%)*	718 (42.3)
≥ 40, *n (%)*	157 (9.2)
Body mass index, kg/m^2^, mean ± SD	24.0 ± 3.0
Body mass index, kg/m^2^	
< 18.5, *n (%)*	51 (3.0)
18.5–24.9, *n (%)*	936 (55.1)
25–29.9, *n (%)*	682 (40.1)
≥ 30, *n (%)*	31 (1.8)
Marital status	
Married, *n (%)*	1453 (85.5)
Not married, *n (%)*	247 (14.5)
Work status	
Working, *n (%)*	1174 (69.1)
Not working, *n (%)*	524 (30.8)
Not applicable, *n (%)*	2 (0.1)
Social/financial status	
High, *n (%)*	81 (4.8)
Medium, *n (%)*	1502 (88.4)
Low, *n (%)*	3 (0.2)
Unknown, *n (%)*	114 (6.7)

*SD* standard deviation

MCD diagnoses and the prescribed treatment are summarized in Table 2. Dysmenorrhea and mastodynia/mastalgia related to PMS were the most common menstrual disorders (43.8% and 21.1%, respectively) followed by menorrhagia or menometrorrhagia (8.8%) and hypomenorrhagia (8.7%). There were also patients who had MCDs in combination with mastodynia/mastalgia (6.7%) or other combinations of MCDs (1.0%). There were no patients with amenorrhea as main disease in this study.

**Table 2:** Menstrual cycle disorders and treatment in the study population

	Cyclodynon® (*n* = 1303)	Mastodynon® (*n* = 363)	Cyclodynon® + Mastodynon® (*n* = 34)	Study population (*N* = 1700)
MCD type				
Dysmenorrhea, *n (%)*	738 (56.6)	3 (0.8)	3 (8.8)	744 (43.8)
Mastodynia/mastalgia related to PMS,* n *(%)	6 (0.5)	352 (97.0)	1 (2.9)	359 (21.1)
Menorrhagia or Menometrorrhagia, *n* (%)	149 (11.4)	0	1 (2.9)	150 (8.8)
Hypomenorrhagia, *n* (%)	147 (11.3)	1 (0.3)	0	148 (8.7)
Intermenstrual bleeding or metrorrhagia, *n *(%)	57 (4.4)	0	0	57 (3.4)
Hypermenorrhagia, *n* (%)	50 (3.8)	0	0	50 (2.9)
Oligomenorrhea, *n* (%)	38 (2.9)	0	1 (2.9)	39 (2.3)
Polymenorrhea, *n* (%)	22 (1.7)	0	0	22 (1.3)
Any MCDs and mastodynia/mastalgia, *n *(%)	79 (6.1)	7 (1.9)	28 (82.3)	114 (6.7)
Combination of MCDs (excluding mastodynia/mastalgia), *n *(%)	17 (1.3)	0	0	17 (1.0)

*MCDs* menstrual cycle disorders

Cyclodynon® was prescribed for 76.7% of patients; dysmenorrhea, hypomenorrhagia, and menorrhagia or menometrorrhagia were the most common indications (56.6%, 11.3%, and 11.4%, respectively). Mastodynon® was prescribed for 21.4% of patients, and the majority of them presented with mastodynia/mastalgia (97.0%). It was found that 2.0% of patients were prescribed treatment with both medications; therefore, it is not possible to attribute the treatment effects to a single medication in these cases. The retrospectively analyzed treatment period was 3 (± 1) months from the date of prescription in almost all patients (99.8%); therapy may have been continued afterward.

The women’s gynecological characteristics are listed in Table 3. An irregular menstrual cycle was reported by 9.1% of women and was mainly associated with patients suffering from a combination of several MCDs, oligomenorrhea or any MCDs and mastodynia/mastalgia (58.8%, 39.5% and 27.9% of patients with irregular cycle, respectively). About 40% of women experienced breast tenderness.

**Table 3:** Gynecological characteristics of the study population

Parameter	Study population (*N* = 1700)
Menarche age, years, mean ± SD (range)	13.2 ± 1.5 (9–18)
Menstrual cycle regularity	
Irregular, *n* (%)	155 (9.1)
Regular, *n* (%)	1545 (90.9)
Menstrual cycle length, days, mean ± SD (range)	29.4 ± 2.8 (12–45)
Duration of menstruation, days, mean ± SD (range)	4.6 ± 0.9 (2–9)
Any gynecological surgeries, *n (%)*	577 (33.9)
Breast abnormalities	
Breast tenderness, *n* (%)	679 (39.9)
Pathologic breast condition (palpation), *n* (%)	108 (6.4)
Breast lesions or/and cysts (ultrasound investigation), *n* (%)	459 (27.0)

*SD* standard deviation

Overall, 38.6% of patients reported the presence of concomitant diseases. The most common diseases included uterine leiomyoma (8.6%), iron-deficient anemia (6.1%), and uterine endometriosis (5.9%). At least one concomitant medication was reported by 16.8% of patients, and iron trivalent oral preparations as well as progestogens and estrogens fixed combinations were the most commonly used drugs (reported by 6.4% and 4.4% of women, respectively). Additional data of gynecological and obstetric characteristics are displayed in Supplementary Table S1.

Baseline assessments of the women’s QoL showed that mood swings and changes in concentration were the most prevalent symptoms (76.6% and 40.6%, respectively). Further prevalent psychologic symptoms were decreased libido, headache, unsatisfactory sleep quality and stomach complaints (29.1%, 29.1%, 24.6% and 22.1%, respectively).

### Improvement of cycle associated complaints

**Fig. 1:**
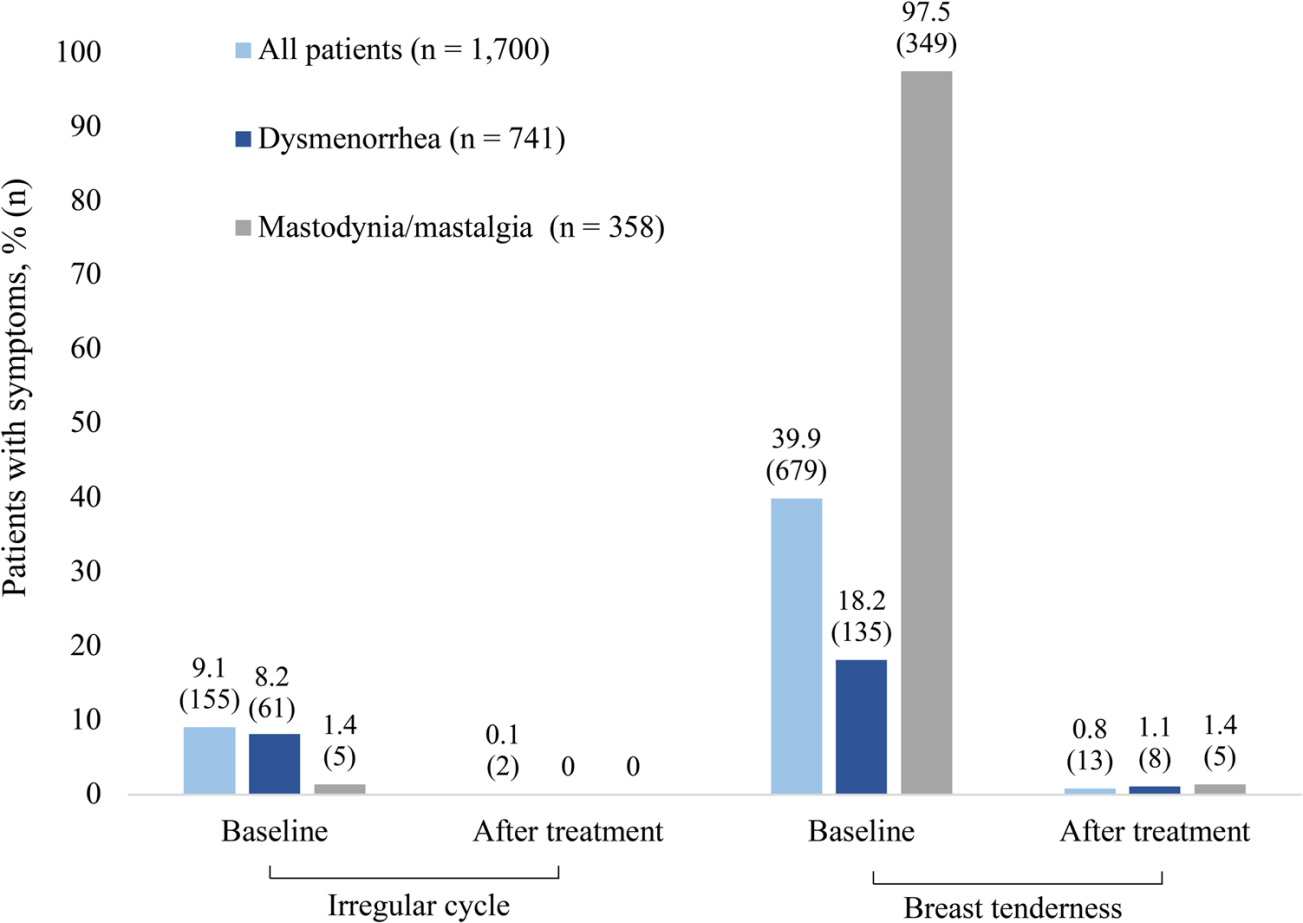
Patients with irregular cycle or breast tenderness before and after treatment by disease category

Treatment with Cyclodynon® and Mastodynon® was associated with a considerable decrease in the percentage of patients with irregular cycle and breast tenderness already after three (± 1) months (Fig. 1). In the full analysis set of all 1700 patients, irregular cycle and breast tenderness decreased from 9.1% to 0.1% and 39.9% to 0.8% of patients, respectively. A strong treatment effect was also seen in the subgroup of patients with dysmenorrhea with a decrease from 8.2% to 0% and 18.2% to 1.1% of patients with irregular cycle and breast tenderness, respectively. Patients with mastodynia/mastalgia benefited greatly from VAC treatment in terms of the presence of breast tenderness: At baseline, 97.5% of patients suffered from breast tenderness, while the proportion decreased to 1.4% after treatment. The proportion of patients with irregular cycle decreased from 1.4% to 0% after treatment. Patients treated with both products were not included in this analysis in order to relate the effects to the specific product.

**Fig. 2:**
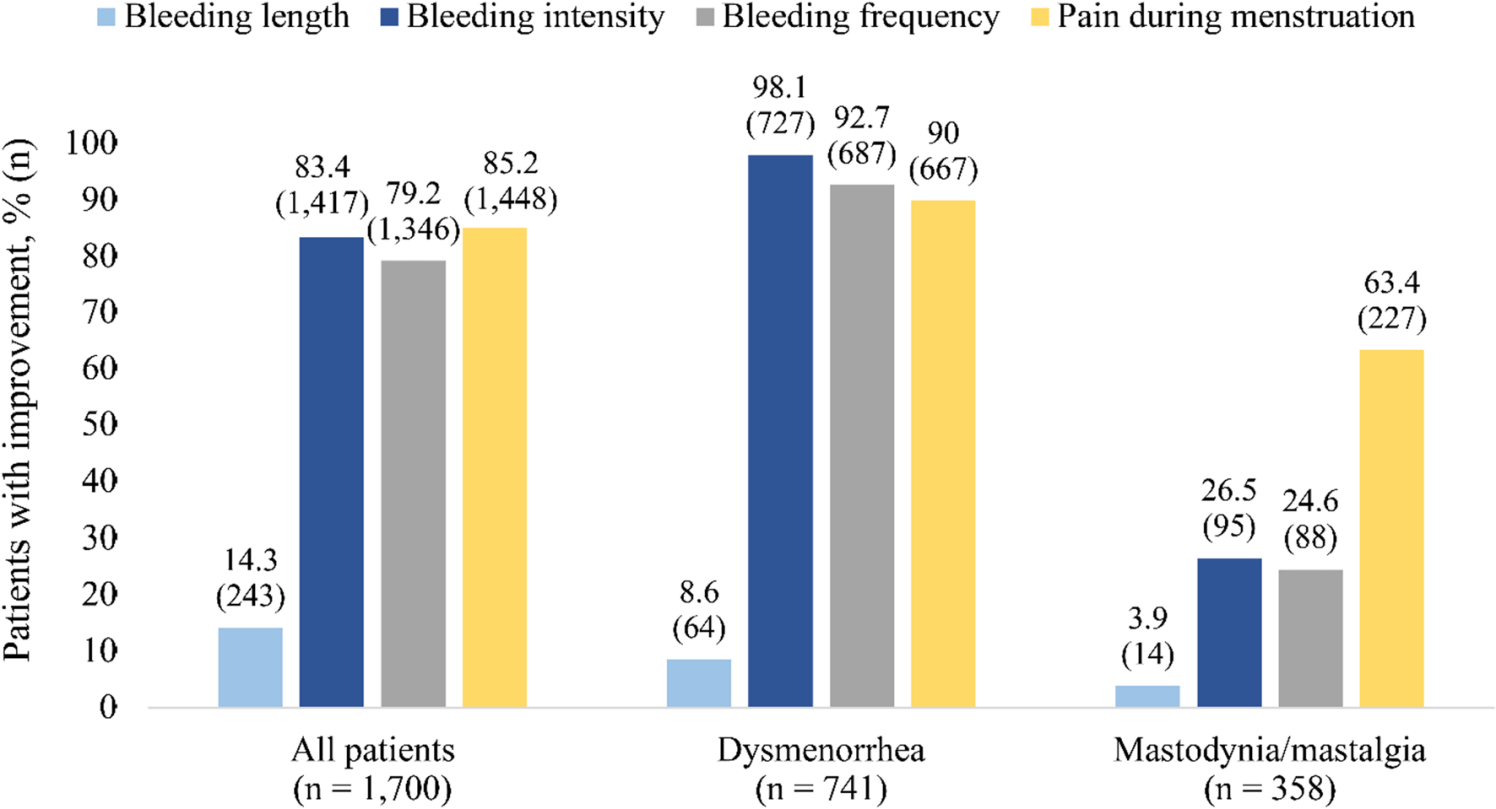
Proportion of patients with improvement in bleeding length, bleeding intensity, bleeding frequency and pain during menstruation after treatment by disease category

Figure 2 shows improvements in bleeding length, bleeding intensity, bleeding frequency and pain during menstruation for all patients, as well as for the subgroups of patients with dysmenorrhea and patients with mastodynia/mastalgia treated with one of the studied VAC products. An improvement in bleeding length, intensity, and frequency was experienced by 14.3%, 83.4%, and 79.2% of all patients, respectively. A considerable number of patients (85.2%) reported improvement in menstrual pain through the observation period. Analysis by disease category revealed that improvement rates were more pronounced for dysmenorrhea patients while in patients with mastodynia/mastalgia generally less pronounced improvement in the evaluated paramters was observed. Variable results were revealed for bleeding length in all disease categories (Supplementary Table S2). Notably, for each symptom, worsening was observed in only one out of 1700 patients (0.1%).

Spontaneous menstruation was documented for two patients with dysmenorrhea. At the follow-up visit, one patient reported additional bleeding episodes and one patient with menorrhagia as main disease reported amenorrhea. No meaningful changes in cycle duration or menstruation duration were observed (mean [SD] change from baseline values were −0.4 [± 1.77] days and −0.1 [± 0.37] days, respectively), BMI also remained unchanged (change from baseline was 0.06 [± 0.32] kg/m^2^).

### Improvement of quality of life

**Fig. 3:**
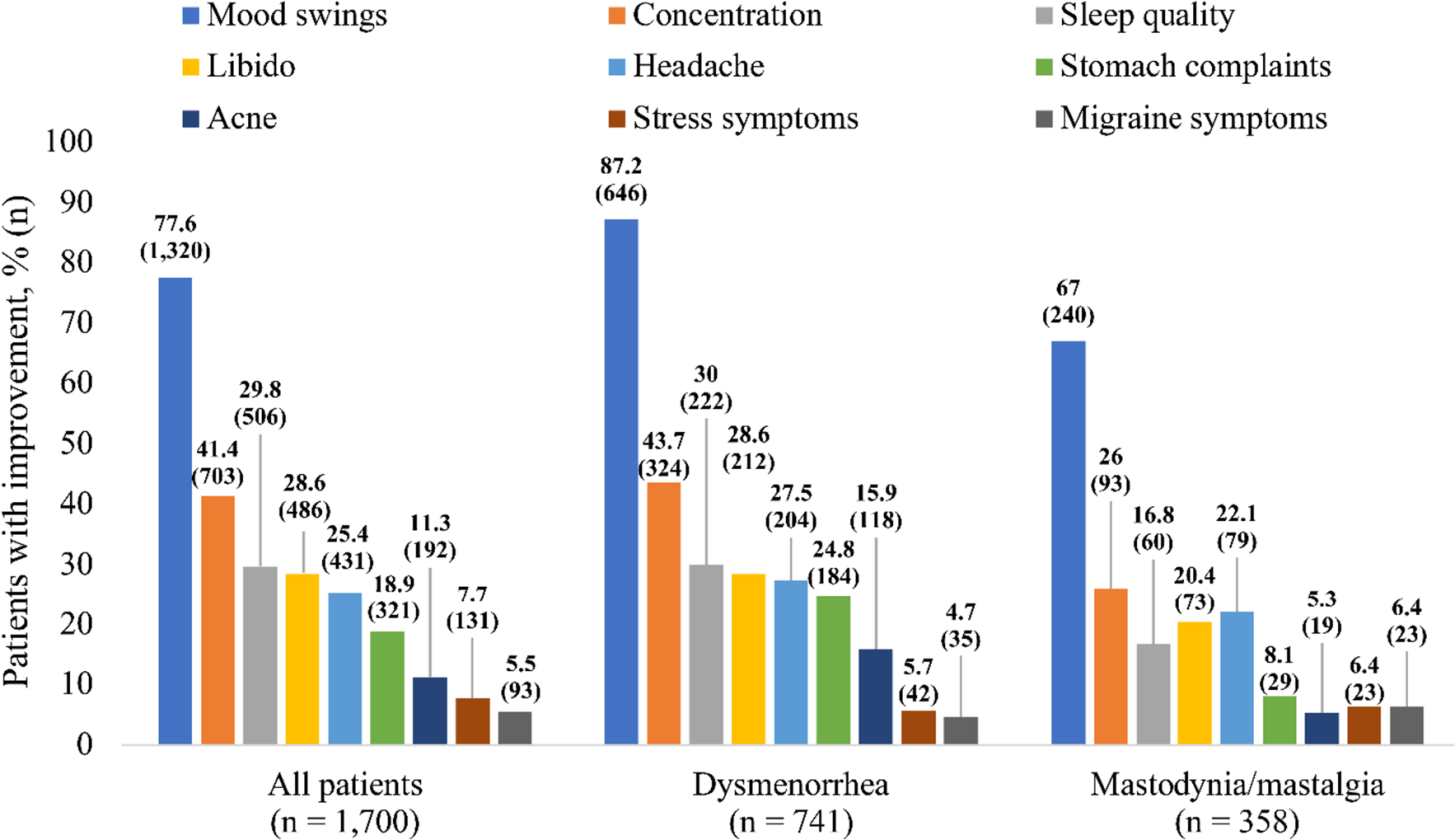
Proportion of patients with improvement in categories of QoL by disease category

Patients with MCDs experienced improvements in all aspects of QoL during the observation period. The most considerable changes were revealed for mood swings with improvement in 77.6% of all patients, 87.2% of patients with dysmenorrhea and 67% of patients with mastodynia/mastalgia (Fig. 3). Substantial improvement was also reported for concentration, sleep quality, headache and stomach complaints. The least improvement was shown for depression symptoms with 2.4% of all patients, 1.9% of patients with dysmenorrhea and 2.5% of patients with mastodynia/mastalgia as well as for constipation with 2.8% of all patients, 4.5% of patients with dysmenorrhea and 1.4% of patients with mastodynia/mastalgia (data not shown).

An additional analysis in the subgroups of women who reported episodes of headache (*n* = 352) or migraine (*n* = 94) or both headache and migraine (*n* = 142) at baseline showed that VAC products had beneficial effects on the QoL. Among women with headache complaints at baseline, an improvement of headache was reported by 85.5% of patients. Women with migraine complaints also benefitted from the VAC treatment: 36.2% of patients reported an improvement of the migraine symptoms. Among women with both headache and migraine complaints at baseline, an improvement of the dynamics of the headache after the end of treatment was reported by 81.0% of these women. All extracted data for QoL are shown in Supplementary Table S3.

### Daily functioning

The number of patients with any period of sick leave of at least one day or reduced daily functioning due to MCDs decreased from 47.5% to 0.6% during the observation period.

## Discussion

The present study is the first to show real-world data of the use of Cyclodynon® and Mastodynon® containing 4 mg of VAC (BNO 1095) to treat different types of MCDs. Treatment with Cyclodynon® and Mastodynon® considerably improved painful menstrual bleeding, breast tenderness and the patient’s QoL, thereby supporting published data [16, 22, 26, 27] and providing proof of their effectiveness in a routine clinical setting. However, the extend of the improvement varied depending on the underlying disease.

In this real-world study the baseline characteristics of the enrolled patients are consistent with known data on the population of women of reproductive age with MCDs [20–23, 26] and demonstrate that VAC products are administered for a wide range of indications in the real-world setting. Most women presented with dysmenorrhea and cyclic mastodynia/mastalgia related to PMS, which is not surprising since these diseases are among the most common menstrual complaints. Baseline assessment of QoL showed that mood swings were most prevalent. Other common complaints were changes in concentration, decreased libido, headache, unsatisfactory sleep quality and stomach complaints. In line with the approved indications, Cyclodynon® was more frequently prescribed for treating dysmenorrhea and AUB and treatment with Mastodynon® was mainly prescribed for patients with breast disorders (mastodynia or mastalgia) related to PMS.

Initiation of the treatment with Cyclodynon® and Mastodynon® was associated with a considerable decrease in the percentage of patients with irregular cycle (from 9.1% to 0.1% of patients) and breast tenderness (from 39.9% to 0.8%) already after three months in this real-world study, a time frame in which studies with hormone preparations also describe an improvement of symptoms [28–31]. A significant number of patients reported improvement in menstrual pain (85.2%), bleeding intensity (83.4%) and frequency (79.2%) during the observation period. Analysis by disease category showed improvements in the beforementioned parameters in patients suffering from dysmenorrhea, mastodynia/mastalgia, a combination of MCDs and mastodynia/mastalgia in combination with any MCDs, which represent the most prevalent MCDs of the women of this study. Strikingly, the decrease in bleeding intensity, frequency and menstrual pain was more pronounced in patients suffering from dysmenorrhea compared to patients suffering from mastodynia/mastalgia as main disease. The potential of VAC to alleviate dysmenorrhea was also shown by a prospective comparative case–control study of Turkish women with severe primary dysmenorrhea, where the efficacy of Cyclodynon®^1^ was comparable to that of ethinyl estradiol/drospirenone in reducing the intensity of menstrual pain [21]. Likewise, the beneficial therapeutic effect of Mastodynon® for treatment of AUBs and dysmenorrhea was shown in a multicenter study with more than 1300 patients. Efficacy and tolerability of Mastodynon® was investigated over the treatment course of three months, with patients receiving 30 drops of Mastodynon® twice daily [20]. Treatment with Mastodynon® led to a normalization of menstrual cycle function and eliminated various menstrual cycle irregularities. Depending on the severity, 62–82% of the cases of menstrual irregularities were normalized [20]. In the same study, VAC proved to be effective for painful menstrual bleeding: In a cohort of 585 patients with dysmenorrhea, treatment with Mastodynon® for three  months led to alleviation of pain symptoms in 66.3% of patients [20].

A recently published systematic review and meta-analysis of RCTs showed that VAC is a safe and effective treatment option for cyclic breast pain [26]. VAC extract was effective in relieving breast pain intensity and lowering the increased serum prolactin level in reproductive age patients (18–45 years) suffering from cyclic breast pain with or without PMS. Moreover, it was shown that in seven of the analyzed studies, VAC was a non-inferior alternative to pharmaceutical therapies for cyclic mastalgia, including dopamine agonists, nonsteroidal anti-inflammatory drugs, serotonin reuptake inhibitors, and hormonal contraceptives. Almost all patients of our real-world study who were prescribed Mastodynon® suffered from mastodynia/mastalgia as main menstrual cycle disease (97.5%), which reflects a prescription in accordance with its approved indication. Importantly, this study also shows the effectiveness of Mastodynon® in the real-world setting, with only 1.4% of patients still suffering from mastodynia/mastalgia after therapy. Of note, a less pronounced improvement in other evaluated parameters such as bleeding intensity and bleeding length was observed in mastodynia/mastalgia patients in this real-world study. However, this could be due to the fact that most patients (98.6%) already had a regular cycle at the beginning of the study.

MCDs can largely impact the everyday life of women. Initiation of therapy with Cyclodynon® and Mastodynon® improved the QoL and everyday-life functioning. The most significant changes were revealed for the parameters mood swings and concentration (77.6% and 41.4% of patients with improvement, respectively). In addition, women experienced improvements of their sleep quality, libido, as well as stomach complaints. The number of patients with periods of sick leave or reduced daily functioning due to MCDs decreased considerably from 47.5% to 0.6% after the course of treatment. Treatment with VAC extract can therefore have positive socio-economic effects. In addition, treatment with Cyclodynon® and Mastodynon® also positively influenced the QoL of women suffering from menstrual headache and/or migraine attacks. More than 80% of these women reported improvement in headache and more than 30% of women had improvement in migraine at the end of the study period. This is in line with the findings of Ambrosini and colleagues: in their open-label clinical observation study with 100 women with migraine and PMS who received Cyclodynon® treatment for a 3-month period, 42% of patients experienced a reduction higher than 50% in frequency of monthly migraine attacks, and 57% of patients experienced a reduction higher than 50% in monthly days with headache [23]. The effect might be explained by the anti-inflammatory properties of VAC and its pharmacological activities on the reproductive steroid, dopaminergic, and opiatergic systems. Cyclodynon® also demonstrated a considerable improvement in the psycho–emotional status of women with primary dysmenorrhea [24]. This result is reflected by our real-world study, which found a greater improvement in QoL parameters in patients suffering from dysmenorrhea compared to mastodynia/mastalgia patients, potentially due to the fact that QoL was already more negatively impacted in the dysmenorrhea group at baseline.

In this study, only four patients aged 12–17 years were included, as these patients are not routinely observed in the selected study center. However, all four patients experienced improvements in bleeding intensity and frequency as well as menstrual pain to a similar extent as in adults (data not shown). This finding is not surprising, since a recent review of 14 studies showed that Cyclodynon® is also effective and safe in female adolescents and young women (aged 12–26) with MCDs and even leads to normalization of prolactin levels [14].

Menstrual disorders such as AUB, dysmenorrhea and mastalgia/mastodynia related to PMS require long-term treatment, therefore good tolerability and low toxicity are very important [32]. In this real-world study, there were no unexpected safety findings. The currently available data also demonstrate that VAC products are safe herbal medicines. Systematic reviews showed that the adverse events following VAC treatment are generally mild and reversible. The most prevalent side effects are nausea, headache, gastrointestinal disturbances, menstrual disorders, acne, pruritus, and erythematous rash [16, 22, 26]. Therefore, a VAC extract is better tolerated than bromocriptine, which has a high rate of adverse events [26].

There are several limitations regarding real-world data in general and this study in particular that should be considered. In general, the retrospective nature of these studies and the routine clinical setting result in limitations such as the inability to obtain complete and verified data, which may compromise the interpretation of study results. In this study in particular, a potential bias might be introduced by the single-center uncontrolled design and subjective nature of the evaluated symptoms. Possible measurement errors cannot be completely excluded, as no validated questionnaires were used. Future studies should therefore integrate validated questionnaires such as the dysmenorrhea daily diary (DysDD) [33] into their study design. In this study, the age distribution was balanced with the exception of adolescent patients (12–17 years 0.2%, 18–24 years 23.1%, 25–29 years 25.2%, 30–34 years 25.5%, 35–39 years 16.8%,  ≥ 40 years 9.2%). Given the fact that MCDs such as dysmenorrhea already occur with onset of menarche [5], a study population including more adolescent patients would be desirable to evaluate the effects of VAC extract throughout the whole lifespan. Finally, the analyzed duration of VAC treatment in the current study was only 3 (± 1) months, therefore future studies are needed to evaluate longer-term real-life effectiveness and safety.

### Supplementary Information

Below is the link to the electronic supplementary material.

Supplementary file1 (DOCX 19 kb)

## Data Availability

Not applicable: Due to data protection laws in Russia only a study report but no raw data are available.

## Footnotes

1 In Turkey, the trade name of the product is Agnucaston®.

## Declarations

The study was conducted in accordance with legal and regulatory requirements, as well as with scientific purpose, value and rigor and follow generally accepted research practices described in Guidelines for Good Pharmacoepidemiology Practices (GPP) issued by the International Society for Pharmacoepidemiology (ISPE), Good Epidemiological Practice (GEP) guidelines issued by the International Epidemiological Association (IEA), Good Practices for Outcomes Research issued by the International Society for Pharmacoeconomics and Outcomes Research (ISPOR), International Ethical Guidelines for Epidemiological Research issued by the Council for International Organizations of Medical Sciences (CIOMS).
