# Supplementary material for: Use of Vitex agnus-castus in patients with menstrual cycle disorders: a single-center retrospective longitudinal cohort study
Source: Arch Gynecol Obstet. 2024 Feb 23;309(5):2089–98. doi: 10.1007/s00404-023-07363-4 (PMC11018691; doi:10.1007/s00404-023-07363-4)
Supplement: Supplementary file 1 — Supplementary file1 (DOCX 19 kb) [file 404_2023_7363_MOESM1_ESM.docx]

**Supplements**

Use of *Vitex agnus-castus* in women with menstrual cycle disorders: a single-center retrospective longitudinal cohort study

**Table S1:** Gynecological and obstetric characteristics of the study population, all data

| Parameter | Study population (*N* = 1,700) |
| --- | --- |
| Number of pregnancies, mean ± SD (range) | 1.9 ± 1.1 (0–8) |
| Number of deliveries, mean ± SD (range) | 1.4 ± 0.8 (0–6) |
| Number of deliveries  0, *n (%)*  1–3, *n (%)*  ≥4, *n (%)* | 202 (11.9)  1488 (87.5)  10 (0.6) |
| Number of abortions, mean ± SD (range) | 0.5 ± 0.7 (0–4) |
| Current method of contraception  Barrier, *n (%)*  Hormonal, *n (%)*  Calendar method, *n (%)*  Interrupted sexual intercourse, *n (%)*  No contraception, *n (%)*  Not applicable (sterility), *n (%)* | 1,098 (64.6)  82 (4.8)  233 (13.7)  181 (10.6)  102 (6.0)  4 (0.2) |
| Sterility, *n (%)* | 5 (0.3) |
| Previous use of assisted reproductive technologies, *n (%)* | 1 (0.1) |

**Table S2:** The proportion of patients with improvement in menstrual bleeding and pain by disease category

| Disease category | Patients with improvement, *n (%)* | | | |
| --- | --- | --- | --- | --- |
|  | Bleeding length | Bleeding intensity | Bleeding frequency | Pain during menstruation |
| Dysmenorrhea (*n* = 741) | 64 (8.6) | 727 (98.1) | 687 (92.7) | 667 (90.0) |
| Mastodynia/mastalgia (*n* = 358) | 14 (3.9) | 95 (26.5) | 88 (24.6) | 227 (63.4) |
| Intermenstrual bleeding or Metrorrhagia (*n* = 57) | 27 (47.4) | 57 (100) | 55 (96.5) | 55 (96.5) |
| Hypomenorrhagia (*n* = 148) | 20 (13.5) | 144 (97.3) | 143 (96.6) | 132 (89.2) |
| Menorrhagia or Menometrorrhagia (*n* = 149) | 38 (25.5) | 148 (99.3) | 136 (91.3) | 134 (89.9) |
| Hypermenorrhagia (*n* = 50) | 10 (20.0) | 50 (100) | 45 (90.0) | 45 (90.0) |
| Oligomenorrhea (*n* = 38) | 17 (44.7) | 38 (100) | 37 (97.4) | 30 (78.9) |
| Polymenorrhea (*n* = 22) | 9 (40.9) | 21 (95.5) | 21 (95.5) | 21 (95.5) |
| Any MCD and mastodynia/mastalgia (*n* = 86) | 17 (19.8) | 86 (100) | 83 (96.5) | 86 (100) |
| Combination of MCDs (excluding mastodynia/mastalgia) (*n =* 17) | 10 (58.8) | 17 (100) | 17 (100) | 17 (100) |

*^MCD^* ^menstrual cycle disorder^

**Table S3:** Changes in quality of life after treatment

|  | Study population (*N* = 1700) | | | |
| --- | --- | --- | --- | --- |
|  | Improvement | Worsening | No change | Reported as  not applicable |
| Sleep quality, *n (%)* | 506 (29.8) | 2 (0.1) | 1,191 (70.1) | 1 (0.1) |
| Stress symptoms, *n (%)* | 131 (7.7) | 1 (0.1) | 1,566 (92.1) | 2 (0.1) |
| Migraine symptoms, *n (%)* | 93 (5.5) | 2 (0.1) | 1,603 (94.3) | 2 (0.1) |
| Headache, *n (%)* | 431 (25.4) | 2 (0.1) | 1,266 (74.5) | 1 (0.1) |
| Acne, *n (%)* | 192 (11.3) | 0 (0.0) | 1,504 (88.5) | 4 (0.2) |
| Constipation, *n (%)* | 47 (2.8) | 3 (0.2) | 1,646 (96.8) | 4 (0.2) |
| Stomach complains, *n (%)* | 321 (18.9) | 0 (0.0) | 1,375 (80.9) | 4 (0.2) |
| Changes in concentration, *n (%)* | 703 (41.4) | 1 (0.1) | 995 (58.5) | 1 (0.1) |
| mood swings, *n (%)* | 1320 (77.6) | 0 (0.0) | 379 (22.3) | 1 (0.1) |
| Depression symptoms, *n (%)* | 41 (2.4) | 0 (0.0) | 1,656 (97.4) | 3 (0.2) |
| Libido, *n (%)* | 486 (28.6) | 8 (0.5) | 1,205 (70.9) | 1 (0.1) |
